# Genome Informatics 2014

**DOI:** 10.1186/s13059-014-0543-7

**Published:** 2014-11-22

**Authors:** Jared T Simpson

**Affiliations:** Ontario Institute for Cancer Research, Toronto, M5G 0A3 Canada

## Abstract

A report on Genome Informatics 2014, held in Cambridge, UK, 21-24 September, 2014.

The first time I attended Genome Informatics was in 2010. The content of the talks and posters reflected a field that was still coming to terms with the era of short read sequencing. Many presentations were devoted to the analytical challenges of working with high-throughput sequencing data; assembly, alignment, variant calling and data storage were frequent topics both formally from the podium and informally in the hallways. The latest Genome Informatics meeting, held at Churchill College in Cambridge, UK, 21-24 September 2014, highlighted just how far the field has come in only a few years. The focus of the meeting is shifting towards the challenges of applying sequencing at all scales - from individual cells to populations - in all areas of biology. To demonstrate the diverse state of the field, I highlight representative talks from this year’s Genome Informatics conference below.

## Personal and medical genomics

The meeting opened with a series of talks on Personal and Medical Genomics. Atul Butte (Stanford University, USA) gave an overview of his work in translating biomedical discoveries into clinical use. Butte highlighted the value in mining published scientific literature for information on SNPs, phenotypes and diseases but pointed out that the information is often incomplete or conflicting between reports. The difficulty of using incomplete and occasionally inaccurate publications to predict the effect of sequence variation was reiterated by Elizabeth Worthey (Medical College of Wisconsin, USA) later in the session, who reported on her experiences using sequencing to diagnose pediatric disease.

Konrad Karczewski (Massachusetts General Hospital, USA) described how humans harboring loss-of-function (LoF) variation can be thought of as naturally occurring ‘knockout experiments’. Karczewski described analysis tools to improve the quality of LoF variant calls and how large sequenced cohorts can be mined to reveal the function of the disrupted genes.

In recent years there have been many notable success stories in using sequencing to diagnose rare Mendelian diseases. Often though, multiple cases are needed to pin down the causative variant. Orion Buske (The Hospital for Sick Children and the University of Toronto, Canada) presented PhenomeCentral, a system to match individuals afflicted with a rare disease by phenotypic similarity. This work relies on the development of a rich ontology to describe the clinical manifestation of a disease, a topic that was addressed by Peter Robinson (Charité-Universitätsmedizin Berlin, Germany) later in the week.

Whole genome sequencing is increasingly being used to guide personalized therapy for cancer patients with advanced malignancies. Yaoqing Shen (Genome Sciences Centre, BCCA, Canada) described a project to sequence the genome and transcriptome of cancer patients, which are mined for potential therapeutic interventions. Shen indicated that the project is now entering a second phase with plans to analyze the genomes of 5,000 patients.

## RNA, the non-coding genome and epigenomics

Two sessions addressed how sequencing the genome, transcriptome and epigenome can be used to better understand the cell’s regulatory machinery and interpret genomic function. For common diseases, large genome-wide association studies uncover dozens to hundreds of risk loci. Davis McCarthy (University of Oxford, UK) is using whole genome sequencing data to assess the contribution of different variant classes to variance in type 2 diabetes liability. McCarthy’s work indicates a substantial proportion of variance in liability is explained by variants in enhancers, which tissue-based analysis shows is driven by enhancers active in pancreatic islet cells.

Rafael Irizarry (Harvard University, USA) provided striking examples of how confounding variables can distort the interpretation of RNA-Seq and methylation studies. Irizarry’s talk underscored how careful experimental design is crucial for the interpretation of high-throughput experiments. Michael Hoffman (Princess Margaret Cancer Centre, Canada) described analysis tools for cytosine methylation data sets, including methods to integrate results across multiple experiments and predict the effect of methylation on transcription factor binding.

Roderic Guigo (Centre for Genomic Regulation, Spain) delivered one of the conference’s keynote lectures. Guigo discussed early findings from the Genotype-Tissue Expression project (GTEx), which explores transcriptional variation across tissues and individuals. The project includes data from hundreds of individuals and thousands of RNA-Seq samples, which reveal patterns of tissue-specific expression and splicing. Interpreting human transcriptomes was also the subject of talks by Tuuli Lappalainen (New York Genome Center, USA) and Stephen Montgomery (Stanford University, USA). Lappalainen described her work characterizing nonsense-mediated decay and parental imprinting leading to monoallelic expression. Montgomery used transcriptome sequencing of a large family to assess the role of rare non-coding variants in the regulation of gene expression and splicing.

Single cell RNA-Seq provides unprecedented resolution of transcriptional heterogeneity. John Marioni (European Bioinformatics Institute, UK) demonstrated that single cell expression data could be mapped into a spatial context by first building a ‘gene expression atlas’ using *in situ* hybridization of a small number of markers. Maroni used this technique to explore expression patterns in the brain of *Platynereis dumerilii*.

## Algorithms, data structures and databases

A recurring algorithmic topic at the meeting was developing methods that operate on populations. This was best exemplified by Ryan Layer’s talk (University of Virginia, USA), which described a compression-based system for efficiently storing and querying genotypes for millions of samples. Zamin Iqbal (University of Oxford, UK) and I described how assembly graphs can be used to explore and catalog sequence variation in pathogens, human populations and cancers. Jean Monlong (McGill University, Canada) is using patterns of sequence variation across human populations to improve the resolution of structural variation detection.

Serafim Batzoglu (Stanford University, USA) delivered a keynote discussing fast methods for detecting identity-by-descent in large cohorts and improvements to variant calling and imputation methods for low-coverage sequencing of populations. Batzoglou also presented algorithms for reconstructing tumor lineages in cancer and methods to phase sequence variants using barcoded pools of long DNA fragments.

The human reference genome has expanded to incorporate additional haplotypes and novel sequence, improving its representation of known human variation. The most recent update to the reference genome was the topic of a presentation by Valerie Schneider (National Center for Biotechnology Information, USA).

We continue to accumulate vast amounts of biological data. Integrating, storing and making this data available to the research community were the focus of a database session. Gary Saunders (European Bioinformatics Institute, UK) presented Ensembl Variation, a repository of genetic variation for all species. The Ensembl Regulation project, described by Daniel Zerbino (European Bioinformatics Institute, UK), annotates the human genome by collating and integrating experimental data sets, such as ChIP-Seq, DNase-Seq and transcription factor binding assays.

## Emerging technology and new applications of sequencing

The Oxford Nanopore MinION system was recently made available through an early access program. As expected, there was much interest in hearing the experiences of the laboratories participating in the program. James Gurtowski (Cold Spring Harbor Laboratory, USA) described their first few months with the system. Gurtowski reported very long read lengths but with a high error rate when compared to other platforms. However, Gurtowski was able to generate a very contiguous assembly of *Saccharomyces cerevisiae* when combining Oxford Nanopore reads with Illumina data. Nick Loman (Birmingham University, UK) highlighted the small form-factor and low cost of the MinION as key features. Loman presented whole-genome data from *Escherichia coli* K-12 MG1655 generated on the MinION and discussed the effect of controlling the rate of DNA translocation on read quality. Loman also demonstrated the real-time functionality of the MinION to identify bacterial strains in a recent hospital outbreak. Species-level information could be recovered within 20 minutes, serotyping within an hour, and assignment to an outbreak cluster within 2 hours.

The Pacific Biosciences sequencing instrument, which has become prominent for producing excellent assemblies of numerous genomes, was used for two challenging sequencing projects. Samarth Rangavittal (Pennsylvania State University, USA) assembled the gorilla Y chromosome using PacBio data. Pin Tong (University of Edinburgh, UK) applied PacBio sequencing to three *Schizosaccharomyces* species to study centromere evolution in fission yeast.

Two talks presented unconventional applications of sequencing. Janet Kelso (Max Planck Institute for Evolutionary Anthropology, Germany) described how sequencing ancient samples - Neanderthals in Europe and western Asia, Denisovans in southern Siberia and a 45,000-year-old modern human - provides insight into the relationship between modern humans and archaic groups. Aaron Darling (University of Technology Sydney, Australia) has adopted the Hi-C protocol, which is typically used to assay the three-dimensional structure of genomes, to improve metagenomic assemblies. Metagenomic Hi-C provides information on which sequences are physically close together in the sample; Darling demonstrated how this information can be used to group assembled contigs together into nearly complete species-level reconstructions.

This year’s edition of Genome Informatics highlighted the rapid advances the community has made in applying genomics in all areas of biology, and the increasing applications of sequencing towards questions of human health. These advances are made possible by the development of algorithms, software, instruments, databases and, crucially, partnerships between biologists, clinicians and informaticians. Genome Informatics continues to bring these communities together to explore the future of genomics as it rapidly unfolds.

